# Seroprevalence, seroconversion and seroreversion of *Borrelia burgdorferi-*specific IgG antibodies in two population-based studies in children and adolescents, Germany, 2003 to 2006 and 2014 to 2017

**DOI:** 10.2807/1560-7917.ES.2023.28.34.2200855

**Published:** 2023-08-24

**Authors:** Stefanie Böhm, Tom Woudenberg, Klaus Stark, Merle M Böhmer, Katharina Katz, Ronny Kuhnert, Martin Schlaud, Hendrik Wilking, Volker Fingerle

**Affiliations:** 1 Bavarian Health and Food Safety Authority, Munich, Germany; 2 Postgraduate Training for Applied Epidemiology (PAE), Robert Koch Institute, Berlin, Germany; 3 ECDC Fellowship Programme, Field Epidemiology Path (EPIET), European Centre for Disease Prevention and Control (ECDC), Stockholm, Sweden; 4 Infectious Disease Epidemiology and Analytics Unit, Department of Global Health, Institut Pasteur, Paris, France; 5 Department for Infectious Disease Epidemiology, Robert Koch Institute, Berlin, Germany; 6 Institute of Social Medicine and Health Systems Research, Otto-von-Guericke-University, Magdeburg, Germany; 7 Department for Epidemiology and Health Monitoring, Robert Koch Institute, Berlin, Germany; 8 German National Reference Centre for Borrelia, Oberschleißheim, Germany; * These authors contributed equally to the work and share the last authorship

**Keywords:** *Borrelia burgdorferi*, antibodies, seroprevalence, Lyme disease, Lyme borreliosis, ticks, Germany

## Abstract

**Background:**

Lyme borreliosis (LB), caused by *Borrelia burgdorferi* (*Bb*), is the most common tick-borne infection in Germany. Antibodies against *Bb* are prevalent in the general population but information on temporal changes of prevalence and estimates of seroconversion (seroincidence) and seroreversion are lacking, especially for children and adolescents.

**Aim:**

We aimed at assessing antibodies against *Bb* and factors associated with seropositivity in children and adolescents in Germany.

**Methods:**

We estimated seroprevalence via two consecutive cross-sectional surveys (2003–2006 and 2014–2017). Based on a longitudinal survey component, we estimated annual seroconversion/seroreversion rates.

**Results:**

Seroprevalence was 4.4% (95% confidence interval (CI): 3.9–4.9%) from 2003 to 2006 and 4.1% (95% CI: 3.2–5.1%) from 2014 to 2017. Seroprevalence increased with age, was higher in male children, the south-eastern regions of Germany and among those with a high socioeconomic status. The annual seroconversion rate was 0.3% and the annual seroreversion rate 3.9%. Males were more likely to seroconvert compared with females. Low antibody levels were the main predictor of seroreversion.

**Conclusion:**

We did not detect a change in seroprevalence in children and adolescents in Germany over a period of 11 years. Potential long-term changes, for example due to climatic changes, need to be assessed in consecutive serosurveys. Seroconversion was more likely among children and adolescents than among adults, representing a target group for preventive measures. Seroreversion rates are over twice as high in children and adolescents compared with previous studies among adults. Thus, seroprevalence estimates and seroconversion rates in children are likely underestimated.

**Key public health message**

**What did you want to address in this study?**

Tick-borne Lyme borreliosis is a widespread disease in Europe, caused by bacteria of *Borrelia burgdorferi*. We tested blood samples of children and adolescents from 2014 to 2017 in Germany for *Borrelia burgdorferi*-specific antibodies indicating a previous infection and compared our results to findings from 2003 to 2006. We wished to determine changes over time after a decade: new infections and loss of antibodies.

**What have we learnt from this study?**

We did not see a change in *Bb* antibodies 2003–2006 to 2014–2017 (4.4% and 4.1%). Infections with *Borrelia burgdorferi* were common throughout Germany, especially amongst males and in Southern Germany. In many, antibodies were no longer detectable when retested 11 years later.

**What are the implications of your findings for public health?**

As infections with *Borrelia burgdorferi* can lead to severe disease, our study has a significant public health relevance. This, as well as long-term trends, which may be affected by climatic changes, should be addressed in future studies. Furthermore, our study indicates that *Borrelia burgdorferi* infections continue to be common among children and adolescents and are likely underestimated, which underlines the need for prevention campaigns.

## Introduction

Lyme borreliosis (LB) is a bacterial infection caused by spirochaetes belonging to the *Borrelia burgdorferi* sensu lato *(Bb*) genospecies complex. Lyme borreliosis is the most common tick-borne disease in Europe [1,2], and *B. afzelii* and *B. garinii* are the predominant species causing LB [3,4]. *Borrelia* are transmitted through bites of ticks of *Ixodes* species, in Germany mainly *Ixodes ricinus*.

Symptoms of LB may appear days to months, in rare cases even years, after the tick bite. The skin manifestation erythema migrans (EM) is by far the most common clinical form and occurs within 3 days up to several weeks after a bite [2,5,6]. More severe forms, such as acute neuroborreliosis (NB) or Lyme arthritis (LA), occur at a progressed stage [6-8]. Antibiotic treatment usually results in full recovery [3,6,8,9]. There is currently no approved vaccine for humans [1,6]. A previous infection with *Bb* does not provide reliable immunity and multiple courses of LB may occur [3,6]. Lyme borreliosis is mainly prevented by avoiding tick bites through individual protection measures, such as wearing long and light-coloured clothing, avoiding going through bushes or tick-infested areas, remaining on walkways when in nature or using repellents, as well as by correct early removal of ticks [1,10].

Yearly incidences of reported cases of LB per 100,000 inhabitants vary widely in Europe (from 0.5 in Ireland to 300 in Austria) [11] and also between the 214 German districts that report cases (0.5 to 138) [5]. In nine of the 16 German federal states, EM, NB and LA are mandatorily notifiable. Between 2013 and 2017, the LB incidence fluctuated between 26 and 41 cases per 100,000 inhabitants in Germany, without an apparent trend [5]. The incidence of reported LB cases follows a bimodal age distribution, peaking in children aged 5–9 years, especially in males, and in adults aged 60–69 years, especially in females [5]. The incidences of cranial nerve palsy and meningitis, two severe forms of LB, are higher in children than in adults and highest in the age group of 5–9 years compared with 20–29 years (incidence rate ratio (IRR) = 12.8 and IRR = 14.1) [5]. Furthermore, in 2019, physicians had diagnosed 128,177 LB cases in Germany using the International Classification of Diseases (ICD) code A69.2 [12], corresponding to an incidence of 179 per 100,000 inhabitants. The estimated incidence varied strongly between districts [13].

Seroprevalence estimates in population-based surveys provide more unbiased information about the exposure to *Bb* compared with surveillance data from notification systems, although prevalence of antibodies is not equivalent to clinical disease, as infections often are asymptomatic [4,14,15]. Consecutive serosurveys, preferably over a period of time, can help revealing trends and identifying risk groups. In Germany, a seroprevalence of 4% was measured in children in the period 2003 to 2006 [16] and 9% in adults in 1997 to 1999 and in 2008 to 2011 [17]. To assess the incidence of *Bb* infections in a population, however, repeated measurements of the same individuals are necessary to determine a seroconversion.

Estimates of seroprevalence and seroincidence are useful for prioritisation of public health interventions. They help to assess changes in the risk of acquiring *Bb* infections. Furthermore, seroprevalence estimates serve as a basis to account for pre-test probabilities of serological tests in the context of clinical diagnoses of LB in children and adolescents.

Here, we aimed at analysing and comparing the seroprevalence of *Bb* IgG antibodies among children and adolescents in Germany in two nationwide surveys conducted 11 years apart, identifying factors associated with seropositivity, seroconversion and seroreversion and estimating their annual rates based on longitudinal data of individuals.

## Methods

### Study procedures and description

The first cross-sectional survey (KiGGS Baseline) of the German Health Interview and Examination Survey for Children and Adolescents (KiGGS) of 0–17-year-olds residing in Germany was conducted from 2003 to 2006, the second survey (KiGGS Wave 2) from 2014 to 2017. In both studies, the enrolment was based on a two-step stratified, probability-clustered sampling approach. In the first step, a specified number of study locations was chosen, stratified by federal state and structural factors, with sampling probability proportional to 0–17-year-olds in the population. In the second step, a predefined number of participants per birth cohort, dependent on community size, was randomly selected within the study locations based on registry data. Comprehensive overviews of the study procedures, including sampling strategies, are available elsewhere [18].

A questionnaire study was included: the study participants or the parents of children 10 years or younger responded. There were questions on sociodemographic facts (sex, age, place of residence, population size of the place of residence, socioeconomic status (SES)), leisure time activities (media consumption, physical activity), animal contact (presence of pets in household) and migration background; we append a description of the variables and categorisation in the Supplement. Information on previous LB was not available. A physical examination and testing, including blood sampling, was done for children from the age of 1 year on in KiGGS Baseline and for a representative subset of children from the age of 3 years in KiGGS Wave 2.

To compare seroprevalence estimates of KiGGS Baseline [16] with KiGGS Wave 2, we used samples of 3–17-year-olds. Altogether, 17,640 children participated in KiGGS Baseline and 15,023 in the cross-sectional part of KiGGS Wave 2 (including children from whom blood samples were not available). Blood samples were available from 11,626 (65.9%) of the 17,640 KiGGS Baseline participants and from 2,891 (19.2%) of the 15,023 KiGGS Wave 2 participants.

Simultaneously to the conduct of the cross-sectional KiGGS Wave 2 survey, another blood sample was taken from former KiGGS Baseline participants, providing a longitudinal follow-up (KiGGS follow-up). We included 4,016 (37.0%) of the 10,853 KiGGS follow-up participants from whom blood samples were available to determine the *Bb* serostatus in both studies. In the Supplement, Figure S4 and S5 give an overview of survey and study participants.

### Laboratory methods

Serum samples were tested for IgG at the National Reference Centre for *Borrelia* using same guidelines, assays and approach for all study groups (including the studies in adults [17]), which is also the standard for clinical diagnostics [6,8,19]. We used a two-step approach involving a screening with an enzyme-linked immunosorbent assay (ELISA) (Enzygnost Lyme link VlsE/IgG, Siemens Healthcare Diagnostics GmbH, Eschborn, Germany), followed by a confirmatory immunoblot (line blot) (Borrelia Europe plus TpN17 LINE, IgG, Virotech, Rüsselsheim, Germany) test in case of a positive or borderline ELISA result. The immunoblot test covered a range of specific *Bb* antigens and was considered positive if reactive to at least two bands. Potential cross-reactions were accounted for by including the antigen TpN17, testing a reaction specific to *Treponema pallidum* (causative agent of syphilis), the most relevant potential cross-reaction in Germany. More details on the test systems and test result categorisation are provided in the Supplement. Serum samples that tested either positive or borderline with the ELISA and positive with the immunoblot were considered seropositive. We refer to Supplementary Figure S1 for a scheme displaying this categorisation.

### Statistical analyses

#### Seroprevalence and predictors of seropositivity

We used Pearson’s chi-squared test to test associations for categorical data and t-test for continuous data. We calculated two-sided p values and considered results statistically significant at a threshold of < 0.05. For all analyses, we used the statistical software R (version 4.2.1) [20]. Visualisations were realised using the R-package *ggplot2*. To improve representativeness, we adjusted for the clustered study design and applied study-specific weights [18,21], using the R-package *survey*. We assessed associations with seropositivity calculating odds ratios (OR) and 95% confidence intervals (CI), using univariable logistic regression. Variables for multivariable analyses were selected from available study variables. Relevant variables were identified by a priori selection based on biological and epidemiological plausibility, and on associations found in scientific literature. We refer to Supplementary Table S1 for a list of hypothesised causal relationships and to Supplementary Figure S2 for the corresponding directed acyclic graph (DAG), for which we used the webpage tool *dagitty*. To determine independent predictors of seropositivity, we estimated total effects of exposures of interest and based multivariable models on the DAG; detailed adjustment sets for seropositivity and seroconversion are appended in Supplementary Table S2. Total effects include both the direct effect of an exposure of interest and indirect effects through mediating factors, while adjusting for potential confounders. If no need for adjustment was indicated to test the effect of an individual variable on seropositivity based on the DAG, controlling for additional variables would possibly lead to over-adjustment, and the univariate analysis was considered sufficient. We estimated an age-standardised seroprevalence for KiGGS Wave 2, using the age distribution of participants in KiGGS Baseline, to account for potential effects of age on seroprevalence estimates due to changes in the age distribution of the German population between 2004 and 2015.

#### Seroconversion and seroreversion

We refer to seroconversion if participants tested seronegative in KiGGS Baseline and seropositive in KiGGS Wave 2. Seroreversion, on the other hand, was defined when a person tested seropositive in KiGGS Baseline and seronegative in KiGGS Wave 2. We refer to Supplementary Figure S5 B for a schematic overview. We assessed potential associations with seroconversion and seroreversion calculating ORs and 95% CIs, using univariable logistic regression models, respectively. For multivariable logistic regression, we included characteristics based on our a priori considerations, additionally including immune response at KiGGS Baseline in case of seroreversion. More details for both are appended to this manuscript in Supplementary Figures S2 and S3. Variables prone to change over time, such as pet ownership or leisure time activities, were only included on availability in KiGGS follow-up data.

#### Annual rates

The annual seroconversion (or seroincidence) rate between the two sampling points was calculated using paired samples, after stratifying by the number of years between sampling, using the following formula on each stratum: p_convyear_ = 1 − (1−p_convyearx_)^1/X^, where p_convyear_ is the estimated probability of seroconverting per year and p_convyearx_ is the observed rate of seroconversion in the *x* years between the two time points of sampling. We assumed a constant rate of infection. The seroreversion rate was determined by dividing the total number of seroreversions by the average observation time of participants who tested seropositive in KiGGS Baseline and were resampled in KiGGS Wave 2.

#### Antigen reactivity

Reactivity to the individual antigens included in the immunoblot were read out; for details of the test systems, we refer to the Supplement. Immunogenicity of antigens varies by stage of infection [6,19]. For example, antibodies targeting OspC and VlsE seem to be associated with the early stages of LB, whereas DbpA, p83, p58, and p39 are associated with late stages of LB [3,6,9,19]. Reactions to a broader spectrum of antigens are indicative of an advanced stage of clinical LB at any point in life or even multiple courses of LB but may also be present after (multiple) asymptomatic infection(s) in some persons [6,19]. Thus, band patterns and the number of positive bands are associated with stages of infection [6,9,19]. Among seropositive KiGGS Baseline participants, we assessed if the individual and the cumulative antigen reactions were predictive of seroreversion in univariable logistic regression analysis. All individual antigen reactions were included in the multivariable regression model.

## Results

### Seroprevalence and seropositivity

Of the included 11,626 KiGGS Baseline participants, 511 were seropositive (4.4%, unweighted), and 131 among the 2,891 KiGGS Wave 2 participants (4.5%, unweighted). The overall weighted seroprevalence in children and adolescents aged 3–17 years in Germany was 4.4% (95% CI: 3.9–4.9%) in the period 2003 to 2006 and 4.1% (95% CI: 3.2–5.1%) in 2014 to 2017 (Table 1, Table 2). The age-standardised seroprevalence for KiGGS Wave 2, given the same population distribution as in the KiGGS Baseline, was 4.3%.

**TABLE 1:** Weighted prevalence of *Borrelia burgdorferi-*specific IgG antibodies by different characteristics and results of logistic regression analyses of potential determinants of seropositivity in a cross-sectional survey of children and adolescents (KiGGS Baseline), Germany, 2003–2006 (n = 11,626)

Characteristics	Tested	Seropositive	Prevalence		Univariable analyses		Multivariable analyses^a^	
			%	95% CI	OR	95% CI	aOR	95% CI
Sex								
Female	5,658	212	3.51	2.93–4.08	Reference			
Male	5,968	299	5.2	4.50–5.90	**1.51**	**1.24–1.83**	No adjustment necessary	
Age group (years)								
3–6	2,364	61	2.31	1.58–3.05	Reference			
7–10	3,033	119	4.07	3.23–4.90	**1.79**	**1.24–2.59**	No adjustment necessary	
11–13	2,809	119	3.95	3.08–4.82	**1.74**	**1.19–2.54**		
14–17	3,420	212	6.16	5.26–7.06	**2.77**	**1.94–3.97**		
Region of residence^b^								
Baden-Württemberg	1,347	56	4.09	2.72–5.45	1.13	0.70–1.81	1.11	0.69–1.81
Bavaria	1,474	105	6.97	5.39–8.54	**1.98**	**1.32–2.97**	**1.94**	**1.28–2.93**
Central	1,193	54	4.3	3.43–5.17	1.19	0.81–1.75	1.15	0.76–1.73
North-west	1,459	53	3.65	2.51–4.79	Reference			
North Rhine-Westphalia	2,205	63	2.95	2.14–3.76	0.8	0.52–1.23	0.8	0.52–1.22
East (north)	2,219	94	4.44	3.41–5.48	1.23	0.82–1.84	1.2	0.80–1.81
East (south)	1,729	86	5.79	4.01–7.57	**1.62**	**1.02–2.57**	**1.61**	**1.02–2.54**
Size of municipality^c^								
Rural	2,540	140	6.19	4.94–7.43	**1.91**	**1.29–2.85**	**1.61**	**1.11–2.35**
Town	5,480	262	4.8	4.06–5.55	**1.46**	**1.01–2.12**	1.35	0.96–1.91
Medium-sized town	2,598	78	2.69	2.01–3.37	0.8	0.52–1.22	0.84	0.57–1.23
Metropolitan	1,008	31	3.33	2.25–4.41	Reference			
Socioeconomic status^d^ (244 missing values)								
High	2,755	155	5.46	4.45–6.47	Reference			
Medium	6,856	294	4.39	3.80–4.99	**0.8**	**0.64–0.99**	0.85	0.68–1.07
Low	1,771	46	2.13	1.34–2.92	**0.38**	**0.24–0.58**	**0.5**	**0.32–0.79**
Migration background^e^ (45 missing values)								
Yes	1,751	25	1.38	0.82–1.94	Reference			
No	9,830	483	5.01	4.47–5.55	**3.76**	**2.53–5.59**	Not applicable	
Daily TV consumption (hours) (3,647 missing values)								
0	507	33	5.88	3.71–8.04	Reference			
0.5	2,701	113	3.95	3.00–4.89	0.66	0.41–1.04	0.63	0.39–1.03
1–2	4,122	140	3.32	2.67–3.96	**0.55**	**0.36–0.84**	**0.5**	**0.31–0.80**
3–4	557	10	1.49	0.40–2.57	**0.24**	**0.10–0.56**	**0.26**	**0.11–0.61**
> 4	92	2	1.12	5.26–7.06	**0.18**	**0.04–0.91**	0.23	0.04–1.21
TV consumption on weekends (h/day) (3,772 missing values)								
0	245	19	7.55	3.94–11.16	Reference			
0.5	1,016	39	3.49	2.22–4.77	**0.44**	**0.23–0.85**	**0.41**	**0.22–0.79**
1–2	4,193	156	3.5	2.82–4.18	**0.44**	**0.26–0.77**	**0.35**	**0.19–0.62**
3–4	2,001	67	3.35	2.38–4.33	**0.42**	**0.23–0.80**	**0.32**	**0.16–0.62**
> 4	399	9	1.89	0.51–3.27	**0.24**	**0.09–0.59**	**0.21**	**0.08–0.54**
Weekly media consumption^f^ (622 missing values)								
Low	3,737	196	5.17	4.35–5.98	Reference			
Middle	3,539	167	4.73	3.90–5.57	0.91	0.73–1.14	0.9	0.72–1.12
High	3,728	132	3.57	2.81–4.33	**0.68**	**0.52–0.89**	**0.73**	**0.55–0.97**
Physical activity (only assessed in participants aged 11 years and older; 120 missing values)								
Never	584	19	2.9	1.42–4.38	Reference			
Rarely	320	16	5.39	2.64–8.13	1.91	0.87–4.19	1.48	0.64–3.38
1–2 times per week	1,806	93	5.11	3.95–6.27	1.8	1.00–3.26	1.66	0.92–2.98
3–5 times per week	1,933	120	6.19	5.02–7.37	**2.21**	**1.29–3.79**	**2.1**	**1.24–3.55**
Almost every day	1,466	78	5.34	4.02–6.67	**1.89**	**1.09–3.28**	**1.83**	**1.07–3.13**
Current pet ownership (251 missing values)								
Yes	5,655	286	5	4.33–5.66	**1.34**	**1.09–1.63**	1.21	0.94–1.57
No	5,720	215	3.78	3.16–4.41	Reference			
Cat as a pet (280 missing values)								
Yes	2,220	140	6.48	5.33–7.62	**1.72**	**1.37–2.15**	1.37	1.00–1.86
No	9,126	359	3.88	3.35–4.40	Reference			
Dog as a pet (280 missing values)								
Yes	1,865	89	4.9	3.85–5.96	1.16	0.91–1.47	0.89	0.62–1.29
No	9,481	410	4.27	3.74–4.79	Reference			
Small mammal as a pet (280 missing values)								
Yes	1,700	86	4.87	3.75–5.98	1.14	0.87–1.49	1.22	0.86–1.73
No	9,646	413	4.29	3.75–4.84	Reference			

aOR: adjusted odds ratio; CI: confidence interval; KiGGS: German Health Interview and Examination Survey for Children and Adolescents; OR: odds ratio.

^a^ Multivariable regression models include adjustment variables according to the minimal sufficient adjustment sets listed in Supplementary Table S2.

^b^ Central: Hesse, Rhineland-Palatinate and Saarland; North-west: Bremen, Hamburg, Lower Saxony and Schleswig-Holstein; East (north): Berlin, Brandenburg, Mecklenburg-Vorpommern and Saxony-Anhalt; East (south): Saxony and Thuringia.

^c^ Size of municipality was classified as residential areas based on population sizes and categorized into rural (< 5,000), town (5,000– < 20,000), medium-sized town (20,000-– < 100,000) and metropolitan areas (≥ 100,000).

^d^ Categorisation of socio-economic status was based on an index, which was calculated using information regarding education and occupational qualifications, occupational status and net equivalent income of the parents. More details are given in the Supplement.

^e^ Participants were assigned a migration background if they had moved to Germany and at least one parent was born abroad, if both parents had moved to Germany or if neither parent had a German citizenship.

^f^ Media consumption was categorised as high, middle or low based on an index. More details are given in the Supplement.

Entries in bold represent statistically significant findings.

**TABLE 2:** Weighted prevalence of *Borrelia burgdorferi-*specific IgG antibodies by different characteristics and results of logistic regression analyses of potential determinants of seropositivity in a cross-sectional survey of children and adolescents (KiGGS Wave 2), Germany, 2014–2017 (n = 2,891)

Characteristics	Tested	Seropositive	Prevalence		Univariable analyses		Multivariable analyses^a^	
			%	95% CI	OR	95% CI	aOR	95% CI
Sex								
Female	1,472	49	2.78	1.87–3.70	Reference		No adjustment necessary	
Male	1,419	82	5.44	3.82–7.06	**2.01**	**1.28–3.15**		
Age group (years)								
3–6	567	11	1.35	0.18–2.51	Reference		No adjustment necessary	
7–10	711	25	3.5	1.81–5.19	2.66	0.97–7.30		
11–13	721	40	4.29	2.72–5.86	**3.28**	**1.26–8.60**		
14–17	892	55	6.81	4.56–9.07	**5.35**	**2.10–13.64**		
Region of residence^b^								
Baden-Württemberg	322	14	4.75	2.44–7.07	2.38	0.95–5.97	2.21	0.88–5.55
Bavaria	360	18	5.26	2.70–7.82	2.65	1.05–6.66	2.33	0.90–6.01
Central	313	13	3.06	1.09–5.04	1.51	0.55–4.16	1.41	0.51–3.94
North-west	358	10	2.05	0.51–3.60	Reference			
North Rhine-Westphalia	556	21	4.5	1.95–7.05	2.24	0.85–5.92	2.11	0.80–5.58
East (north)	535	23	4.15	0.98–7.33	2.06	0.68–6.24	1.86	0.60–5.74
East (south)	447	32	6.19	3.46–8.92	3.14	1.28–7.72	2.66	1.09–6.51
Size of municipality^c^								
Rural	595	31	4.56	3.14–5.98	0.88	0.41–1.88	0.8	0.37–1.76
Town	1,377	70	4.74	3.21–6.27	0.92	0.43–1.97	0.87	0.41–1.82
Medium-sized town	617	15	2.13	0.73–3.53	0.4	0.15–1.05	0.35	0.13–0.95
Metropolitan	302	15	5.14	1.80–8.48	Reference			
Socioeconomic status^d^ (98 missing values)								
High	667	30	3.89	2.29–5.48	Reference			
Medium	1,712	85	5.25	3.81–6.69	1.37	0.81–2.32	No adjustment possible	
Low	414	11	1.39	0.30–2.48	0.35	0.15–0.84		
Daily TV consumption (hours) (1,667 missing values)								
0	73	3	2.01	0–4.60	Reference			
< 1	658	25	3.49	1.71–5.26	1.76	0.45–6.92	1.29	0.32–5.27
1–2	334	2	0.67	0–1.78	0.33	0.05–2.29	0.23	0.03–1.55
2–3	117	3	1.89	0–4.52	0.94	0.14–6.43	0.72	0.11–4.60
3–4	34	0	No adjustment necessary					
> 4	8	0						
Weekly media consumption^e^ (232 missing values)								
Low	974	47	3.62	2.35–4.89	Reference			
Middle	607	27	5.84	3.18–8.51	1.65	0.95–2.86	1.06	0.60–1.89
High	1,078	50	3.93	2.60–5.25	1.09	0.67–1.77	0.66	0.39–1.12

aOR: adjusted odds ratio; CI: confidence interval; KiGGS: German Health Interview and Examination Survey for Children and Adolescents; OR: odds ratio.

^a^ Multivariable regression models include adjustment variables according to the minimal sufficient adjustment sets listed in Supplementary Table S2.

^b^ Central: Hesse, Rhineland-Palatinate and Saarland; North-west: Bremen, Hamburg, Lower Saxony and Schleswig-Holstein; East (north): Berlin, Brandenburg, Mecklenburg-Vorpommern and Saxony-Anhalt; East (south): Saxony and Thuringia.

^c^ Size of municipality was classified as residential areas based on population sizes and categorized into rural (< 5,000), town (5,000– <20,000), medium-sized town (20,000–< 100,000) and metropolitan areas (≥ 100,000).

^d^ Categorisation of socio-economic status was based on an index, which was calculated using information regarding education and occupational qualifications, occupational status and net equivalent income of the parents. More details are given in the Supplement.

^e^ Media consumption was categorised as high, middle or low based on an index. More details are given in the Supplement.

Entries in bold represent statistically significant findings.

In both surveys, seroprevalence increased by age (Figure, Table 1 and 2), more in males than in females. More detailed data differentiating between males and females are provided in Supplementary Table S3. The chance of being seropositive was higher in all older age groups compared with the reference group of 3–6-year-olds and odds ratios were highest in the oldest age category of 14–17 years (Table 1 and 2). Overall, males were more likely to be seropositive than females, with ORs of 1.5 and 2 in the respective surveys (Table 1 and 2).

**FIGURE:**
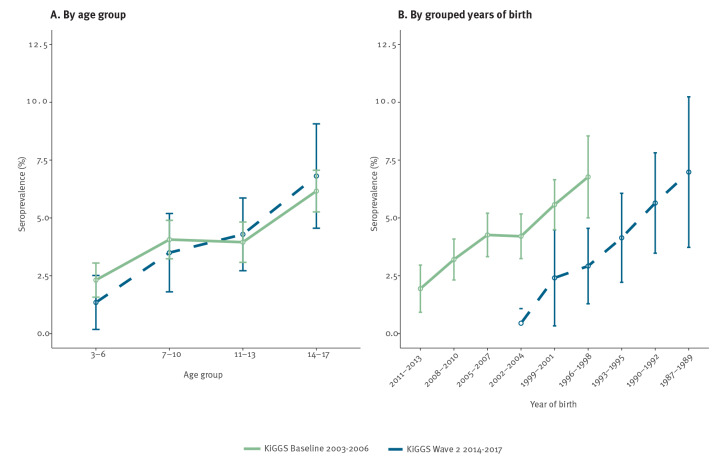
Weighted prevalence of *Borrelia burgdorferi*-specific IgG antibodies in the cross-sectional surveys KiGGS Baseline (n = 11,626) and KiGGS Wave 2 (n = 2,891) of children and adolescents, Germany, 2003–2006 and 2014–2017

KiGGS: German Health Interview and Examination Survey for Children and Adolescents.

In both surveys, the highest seroprevalence was noted in Bavaria and the adjacent federal states Thuringia and Saxony. The prevalence was similar in participants living in medium-sized (20,000– < 100,000 inhabitants) towns (2.7% and 2.1%) but varied to some extent in the metropolitan areas (≥ 100,000 inhabitants) (3.3% vs 5.1%) between the two surveys, although the CIs overlapped. In KiGGS Baseline, children and adolescents living in rural areas (< 5,000 inhabitants) were more likely to be seropositive (OR = 1.6; 95% CI: 1.11–2.35) compared with those living in metropolitan areas. In both surveys, a low SES was associated with reduced odds of being seropositive compared with a high SES (OR = 0.50; 95% CI: 0.32–0.79 and 0.35; 95% CI: 0.15–0.84). More details on the SES are presented in the Supplement. In KiGGS Baseline, the OR of being seropositive was 3.8 (95% CI: 2.53–5.59) for children without migration background vs children with a migration background.

Children and adolescents with a high weekly media consumption were less likely to be seropositive compared with those with low media consumption in KiGGS Baseline (OR = 0.73; 95% CI: 0.55–0.97). Details on the categorisation of media consumption are given in the Supplement. KiGGS Baseline participants who stated being physically active 3–5 times per week had 2.1 times the odds of being seropositive compared with non-active participants. Pet owners in KiGGS Baseline had increased odds of being seropositive compared with children without pets (5.0% vs 3.8%; p < 0.001), particularly cat owners (6.5% vs 3.9%; p < 0.001) (Table 1). In multivariable analysis, the association between cat ownership and seropositivity was not significant. Owning a pet dog or a small mammal was not associated with seropositivity.

### Seroconversion and seroreversion

In the KiGGS follow-up, paired serum samples were available for 4,016 participants, evenly from males (2,052; 51.1%) and females (1,964; 48.9%). Participants were retested after a median of 11 (interquartile range (IQR): 11–11, range: 11–14) years after the KiGGS Baseline study. Most of the 3,885 participants who tested seronegative in KiGGS Baseline were seronegative in KiGGS Wave 2 (3,753; 96.6%); 132 (3.4%) seroconverted. Of the 131 follow-up participants testing seropositive in KiGGS Baseline, 75 (57.3%) were still seropositive in KiGGS Wave 2, while 56 (42.7%) seroreverted. An overview can be accessed in Supplementary Figure S5 B. Being male and of younger age were predictive of seroconversion (Table 3). Median age of seroconverters was 6 years (IQR: 4–10) compared with 8 years (IQR: 4–12) in those who remained seronegative. Living in a rural area was associated with seroconversion compared with living in metropolitan areas, although the CI was wide (Table 3). Children aged 3–6 years in KiGGS Baseline had higher odds for seroreversion than those aged 14–17 years (OR: 4.2; 95% CI: 1.5–12.0). A lower antibody level in the ELISA test in KIGSS Baseline was associated with seroreversion (Table 4). Ten of 11 participants with antibody levels below 10 seroreverted, 39 of 70 with levels between 10 and 99 did so, and 6 of 49 with antibody levels of ≥ 100 seroreverted (Table 4). In the Supplementary Figure S6, a graphical overview is shown.

**TABLE 3:** Predictors of seroconversion of *Borrelia burgdorferi*-specific IgG antibodies among participants of a follow-up to a cross-sectional survey of children and adolescents (KiGGS), Germany, 2003–2006 and 2014–2017 (n = 3,885)

Characteristics	Seronegative participants in KiGGS Baseline		Seroconversions		p value^a^	Univariable analyses		Multivariable analyses^b^	
	n	%	n	%		OR	95% CI	aOR	95% CI
Sex									
Female	1,915	49.3	40	30.3	< 0.01	Reference			
Male	1,970	50.7	92	69.7		**2.30**	**1.59–3.38**	No adjustment necessary	
Age group (years)									
1–2	455	11.7	17	12.9	< 0.01	**2.55**	**1.15–6.03**	No adjustment necessary	
3–6	1,182	30.4	50	37.9		**2.90**	**1.49–6.35**		
7–10	995	25.6	34	25.8		**2.32**	**1.15–5.19**		
11–13	653	16.8	22	16.7		**2.29**	**2.29–5.28**		
14–17	600	15.4	9	6.8		Reference			
Region of residence^c^									
Baden-Württemberg	469	12.1	17	12.9	0.79	1.19	0.59–2.44	1.18	0.58–2.42
Bavaria	509	13.1	21	15.9		1.36	0.70–2.72	1.35	0.69–2.70
Central	393	10.1	11	8.3		0.91	0.40–2.00	0.91	0.40–1.99
North-west	490	12.6	15	11.4		Reference			
North Rhine-Westphalia	792	20.4	23	17.4		0.95	0.49–1.87	0.95	0.50–1.89
East (north)	680	17.5	22	16.7		1.06	0.55–2.10	1.07	0.55–2.12
East (south)	552	14.2	23	17.4		1.38	0.72–2.72	1.36	0.71–2.69
Size of municipality^d^									
Rural	812	20.9	35	26.5	0.04	**2.52**	**1.13–6.72**	**2.39**	**1.04–6.46**
Town	1,854	47.7	70	53.0		**2.20**	**1.03–5.71**	2.13	0.98–5.58
Medium-sized town	877	22.6	21	15.9		1.37	0.58–3.77	1.36	0.57–3.79
Metropolitan	342	8.8	6	4.5		Reference			
Socioeconomic status^e^ (33 missing values)									
High	1,032	26.8	34	25.8	0.72	Reference			
Medium	2,400	62.3	86	65.2		1.09	0.74–1.65	1.08	0.73–1.64
Low	420	10.9	12	9.1		0.86	0.43–1.64	0.82	0.40–1.60
Migration status^f^ (18 missing values)									
Yes	474	12.3	17	12.9	0.82	Reference			
No	3,393	87.7	115	87.1		0.94	0.58–1.64	Not applicable	
Weekly media consumption^g^ (2,305 missing values)									
Low	189	12.0	10	15.6	0.51	Reference			
Medium	423	26.8	14	21.9		0.61	0.27–1.45	0.60	0.26–1.42
High	968	61.3	40	62.5		0.77	0.39–1.66	0.80	0.40–1.75

aOR: adjusted odds ratio; CI: confidence interval; KiGGS: German Health Interview and Examination Survey for Children and Adolescents; OR: odds ratio.

^a^ To compare the proportion of seroconversions, we used the Pearson’s chi-squared test.

^b^ Multivariable regression models include adjustment variables according to the minimal sufficient adjustment sets listed in Supplementary Table S2.

^c^ Central: Hesse, Rhineland-Palatinate and Saarland; North-west: Bremen, Hamburg, Lower Saxony and Schleswig-Holstein; East (north): Berlin, Brandenburg, Mecklenburg-Vorpommern and Saxony-Anhalt; East (south): Saxony and Thuringia.

^d^ Size of municipality was classified as residential areas based on population sizes and categorized into rural (<5,000), town (5,000-<20,000), medium-sized town (20,000-<100,000), and metropolitan areas (≥100,000).

^e^ Categorisation of socio-economic status was based on an index, which was calculated using information regarding education and occupational qualifications, occupational status and net equivalent income of the parents. More details are given in the Supplement.

^f^ Participants were assigned a migration background if they had moved to Germany and at least one parent was born abroad, if both parents had moved to Germany or if neither parent had a German citizenship.

^g^ Media consumption was categorised as high, middle or low based on an index. More details are given in the Supplement.

Entries in bold represent statistically significant findings.

**TABLE 4:** Predictors of seroreversion of *Borrelia burgdorferi*-specific IgG antibodies among participants of a follow-up to a cross-sectional survey of children and adolescents (KiGGS), Germany, 2003–2006 and 2014–2017 (n = 131)

Characteristics	Seropositive participants in KiGGS Baseline		Seroreversions		p value^a^	Univariable analyses		Multivariable analyses^b^	
	n	%	n	%		OR	95% CI	aOR	95% CI
Sex									
Female	47	35.9	19	33.9	0.69	Reference			
Male	84	64.1	37	66.1		1.16	0.56–2.41	Not applicable	
Age group (years)									
1–2	2	1.5	1	1.8	0.10	2.55	0.10–68.29	Not applicable	
3–6	29	22.1	18	32.1		**4.17**	**1.53–12.01**		
7–10	32	24.4	14	25.0		1.98	0.74–5.41		
11–13	29	22.1	12	21.4		1.80	0.65–5.04		
14–17	39	29.8	11	19.6		Reference			
Place of residence^c^									
Baden-Württemberg	17	13.0	5	8.9	0.27	0.54	0.12–2.23	0.53	0.12–2.21
Bavaria	27	20.6	10	17.9		0.76	0.21–2.71	0.82	0.23–2.95
Central	9	6.9	3	5.4		0.64	0.10–3.43	0.65	0.10–3.57
North-west	16	12.2	7	12.5		Reference			
North Rhine-Westphalia	21	16.0	11	19.6		1.41	0.38–5.37	1.40	0.38–5.34
East (north)	22	16.8	14	25.0		2.25	0.61–8.72	2.16	0.58–8.51
East (south)	19	14.5	6	10.7		0.59	0.14–2.36	0.61	0.15–2.42
Size of municipality^d^									
Rural	34	26.0	18	32.1	0.50	1.13	0.23–5.48	1.69	0.31–9.32
Town	70	53.4	27	48.2		0.63	0.14–2.86	0.85	0.17–4.14
Medium-sized town	19	14.5	7	12.5		0.58	0.10–3.18	0.56	0.09–3.33
Metropolitan	8	6.1	4	7.1		Reference			
Socioeconomic status^e^ (2 missing values)									
High	40	31.0	17	30.9	0.84	Reference			
Medium	80	62.0	35	63.6		1.05	0.49–2.29	1.07	0.50–2.33
Low	9	7.0	3	5.5		0.68	0.13–2.96	0.45	0.06–2.24
Antibody level (ELISA)									
< 10	12	9.2	11	19.6	< 0.01	Reference			
10–99	70	53.4	39	69.6		**0.11**	**0.01–0.64**	**0.12**	**0.01–0.78**
≥ 100	49	37.4	6	10.7		**0.01**	**0.00–0.08**	**0.01**	**0.00–0.08**

CI: confidence interval; KiGGS: German Health Interview and Examination Survey for Children and Adolescents; OR: odds ratio; aOR: adjusted odds ratio.

^a^ To compare the proportion of seroreversions, we used the Pearson’s chi-squared test.

^b^ Multivariable regression models include adjustment variables according to the minimal sufficient adjustment sets listed in Supplementary Table S2.

^c^ Central: Hesse, Rhineland-Palatinate and Saarland; North-west: Bremen, Hamburg, Lower Saxony and Schleswig-Holstein; East (north): Berlin, Brandenburg, Mecklenburg-Vorpommern and Saxony-Anhalt; East (south): Saxony and Thuringia.

^d^ Size of municipality was classified as residential areas based on population sizes and categorized into rural (<5,000), town (5,000-<20,000), medium-sized town (20,000-<100,000) and metropolitan areas (≥100,000).

^e^ Categorisation of socio-economic status was based on an index, which was calculated using information regarding education and occupational qualifications, occupational status and net equivalent income of the parents. More details are given in the Supplement.

Entries in bold represent statistically significant findings.

### Annual rates

Observation time for the 4,016 participants was 43,852 person-years. On average, participants contributed 10.9 years (standard deviation (SD): 0.2; range: 10.6–13.8). The average follow-up time was the same in participants with initially seronegative (10.9 years; SD: 0.3) and seropositive (11.0 years; SD: 0.2) test results. The annual seroconversion rate was 0.32% (95% CI: 0.26–0.38), the annual seroreversion rate was 3.91% (95% CI: 3.22–4.78). Eighteen of the 29 initially seropositive children in age group 3–6 years seroreverted whereas 11 of the 39 in age group 14–17 years did so. Although the numbers are small and the CIs are overlapping, we observed decreasing seroreversion rates with increasing age group (Table 5). Average observation times did not differ between age groups.

**TABLE 5:** Association between seroreversion and *Borrelia burgdorferi* antigen-specific IgG antibodies and annual rate of seroreversion by age group among participants of a follow-up to a cross-sectional survey of children and adolescents (KiGGS), Germany, 2003–2006 and 2014–2017 (n = 131)

Characteristics	Seropositive participants in KiGGS Baseline		Seroreversions		p value^a^	Univariable analyses		Multivariable analyses^b^		
	n	%	n	%		OR	95% CI	aOR		95% CI
Antigens (7 missing values)										
OspC	24	19.4	8	16.3	0.49	**0.72**	**0.27–1.80**	**0.55**	**0.16–1.72**	
VlsE	114	91.9	41	83.7	0.006	**0.14**	**0.02–0.59**	0.27	0.04–1.35	
p39	49	39.5	11	22.4	< 0.01	**0.28**	**0.12–0.62**	0.57	0.20–1.60	
p58	76	61.3	19	38.8	< 0.01	**0.20**	**0.09–0.43**	0.60	0.23–1.58	
p83	39	31.5	5	10.2	< 0.01	**0.14**	**0.04–0.36**	0.36	0.10–1.17	
DbpA	99	79.8	30	61.2	< 0.01	**0.14**	**0.05–0.36**	**0.25**	**0.07–0.77**	
Number of antigens (7 missing values)										
1	20	16.1	16	32.7	< 0.01	Reference				
2	23	18.5	13	26.5		**0.33**	**0.07–1.22**	Not applicable		
3	26	21.0	11	22.4		**0.18**	**0.04–0.66**			
4	26	21.0	6	12.2		**0.08**	**0.02–0.29**			
5	21	16.9	3	6.10		**0.04**	**0.01–0.19**			
6	8	6.5	0	0.0		Not applicable				
Age group (years)	n	%	n		p value	Annual rate of seroreversion		95% CI		
1–2	2	1.5	1		0.10	Not applicable				
3–6	29	22.1	18			5.70		5.06–8.52		
7–10	32	24.4	14			4.01		2.57–5.74		
11–13	29	22.1	12			3.81		1.27–3.81		
14–17	39	29.8	11			2.56		0.69–2.78		

aOR: adjusted odds ratio; CI: confidence interval; KiGGS: German Health Interview and Examination Survey for Children and Adolescents; OR: odds ratio.

^a^ To compare the proportion of seroreversions, we used the Pearson’s chi-squared test.

^b^ The multivariable regression model includes all antigen reactions.

Entries in bold represent statistically significant findings.

### Antigen reactivity

Information on antigen-specific antibodies was available for 124 of the 131 follow-up participants who tested seropositive in KiGGS Baseline. Among those, 49 were seroreverters. Considering the individual antigen reactions, the proportion of seroreverters was highest in participants with OspC-specific antibodies (8/24) and lowest among participants with p83-specific antibodies (5/39). In contrast to all other antigens, antibodies targeting the antigen OspC were not significantly associated with seroreversion in the univariable regression analysis (Table 5). In the multivariable regression, including all antigen reactions, only the reaction to DbpA was significantly associated with seroreversion. The number of reactive immunoblot bands in KiGGS Baseline was positively associated with remaining seropositive. Among the 20 participants with one positive band, 16 seroreverted, three of the 21 participants with five reactive bands and none among the eight participants with six reactive bands seroreverted (Table 5); additional figures showing the change in serostatus in relation to the number of bands and antibody levels are appended in Supplementary Figure S6 A.

## Discussion

Comparing seroprevalence data of *Bb* in children and adolescents in Germany based on representative population data, we found no change over the period of a decade. Available for the first time for this age group, serological follow-up 11 years after 1–17-year-olds were first tested, revealed 56 seroreverters among 131 seropositive participants, which was more than we expected.

Similar seroprevalence estimates in the two surveys suggest no change in risk of exposure to *Bb* from 2003 to 2017. This is an interesting finding in light of the discussion about the potential influence of climate change on increases of tick abundance and tick-borne diseases [4,11,22-24]. The interface between climatic factors, ticks, *Borrelia* and hosts is complex, and determinants are poorly understood. The period of 11 years may be too short to detect effects of climate change on exposure to *Bb*. Thus, seroprevalence studies are needed to assess potential long-term effects.

Our estimated seroprevalence was similar to the one reported from Italy (based on ELISA only) [25], but higher than in Sweden [14] and Finland [26] and lower than in Belgium [27]. Differences in seroprevalence across Europe have been described [28], reflecting both differences in *Borrelia* infection rates as well as different study designs and laboratory methods. In Finland, seroprevalence was considerably higher in the late 1960s and early 1970s (20%) [29] than in 2011 (3.9%) [26]. However, due to different laboratory methods, a preponderance of older age groups with no adjustment in the older study and the fact that in the earlier time periods LB had not yet been a known disease with expected lack of early antibiotic treatment, which would have prevented antibody production, comparability of these studies is limited.

In Germany, *Bb* can be found throughout the country, but seroprevalence is heterogeneous between regions. It appears highest in Bavaria and Saxony/Thuringia. Living in a rural area was predictive of seropositivity in KiGGS Baseline, but inconclusive in KiGGS Wave 2. Although many studies have found rural settings to be associated with increased seroprevalence of LB [30,31], more recently, concern has been raised on increasing numbers of tick-borne infections in suburban and urban areas [32]. High infection rates of ticks in urban parks have been reported [33,34] and studies have found comparable seropositivity, LB incidence or risk of LB after a tick bite in urban and rural areas [35,36]. In Hannover, a German city of approximately half a million inhabitants, an increase in the number of *Borrelia*-infected ticks in urban parks was observed, but not in ticks found in forest areas between 2005 and 2015 [33]. However, findings from ticks from one area may not be easily extrapolated to another [33,37].

The increase of seroprevalence by age we found is widely observed [1,17,36]. The estimated annual seroincidence rate in our study was lower than reported in adults (0.32% vs 0.45%), whereas the estimated annual seroreversion rate was more than double compared with another study in adults (3.9% vs 1.5%) [17]. In addition, preschool age (3–6 years) and especially low antibody levels were predictors of seroreversion. This may indicate a less pronounced immune response either due to the evolving immune system in young children or in primary infections, assuming that most infections in the youngest were primary. Repeated exposure leads to higher seroreactivity [38]. Individuals with a previous *Bb*-infection were more likely to experience another infection, as certain risk characteristics remain [39]. Observed seroreversions, especially in the age group of 3–6 years, likely resulted in an underestimation of seroconversions during the follow-up period of 11 years.

We assessed the humoral immunity to *Bb* using serological diagnostic methods routinely used for LB. Seroprevalence studies reported a high proportion of seropositive individuals with no clinical symptoms of LB [4,14,15]. Our representative seroprevalence estimates serve as an important prerequisite to define pre-test probabilities in different age groups. Thus, they guide the interpretation of diagnostic antibody tests (e.g. in an individual with clinical symptoms), as the seroprevalence affects the negative and positive predictive value of diagnostic tests. Similar estimates have already been included in guidelines for clinical diagnoses [6,8] and may prevent misdiagnoses and unnecessary treatment.

Seroreversion rates were dependent on the immune response to *Bb*. Our antigen analyses suggest that participants with signs of a more pronounced immune response remained seropositive for a longer period and were less likely to serorevert. Reactivity to all antigens, except Ospc, was associated with remaining seropositive. Antibodies specific to DbpA were the only predictor of remaining seropositive, independently of the other antigens. This reaction is typically present at a later stage of infection [6,19]. The proportion of seroreverters was highest in participants with specific antibodies to the OspC antigen, a sign of early infection. These early infections are linked to fewer antigen reactions included in the immunoblot, whereas reactions to a broad spectrum of antigens are a sign of infection that occurred long ago [6,19]. Numbers addressing seroreversion, in particular, were small. Still, meaningful conclusions can be derived and these results provide the best evidence base for a population-based assessment of this phenomenon and for the evaluation of *Bb* seropositivity in the population.

Additional testing for *Bb*-specific IgM antibodies may have captured additional early infections, which could have led to higher seroincidence and seroprevalence estimates. Absence of seroconversion in a proportion of asymptomatic or mildly symptomatic (including EM) infected individuals, as well as waning antibody levels over time likely lead to a general underestimation of the actual exposure to *Bb* based on seroprevalence studies, which also applies to our study.

Although small-scale analyses considering diverse ecological or geographical characteristics were not possible with our data, our results still provide differentiation of seroprevalence on a regional scale in one country.

## Conclusion

We found similar seroprevalence estimates in children and adolescents over time. Estimates differed by characteristics such as sex, age or region. We found a high rate of 42% of seroreversions in previously seropositive children. Our findings serve as an important source for further risk assessments, modelling studies and the empirical basis for public health interventions. For example, education should aim to increase awareness in children and their parents as a target group. At the same time, physical and outdoor activities should be promoted, while communicating adequate protection such as tick bite avoidance and correct tick removal. Consecutive serosurveys, preferably with a large proportion of follow-up participants, enable to track risk changes (i.e. due to climate change) over time.

## Supplementary Data

1566000 Supplementary Material
