# Supplementary material for: Seroprevalence, seroconversion and seroreversion of Borrelia burgdorferi-specific IgG antibodies in two population-based studies in children and adolescents, Germany, 2003 to 2006 and 2014 to 2017
Source: Euro Surveill. 2023 Aug 24;28(34):2200855. doi: 10.2807/1560-7917.ES.2023.28.34.2200855 (PMC10451011; doi:10.2807/1560-7917.ES.2023.28.34.2200855)
Supplement: Supplementary Material [file 22-008555_BOHM_Supplement.pdf]

Supplementary Material

This supplementary material is hosted by *Eurosurveillance* as supporting information alongside the article "Seroprevalence, seroconversion and seroreversion of *Borrelia burgdorferi*-specific IgG antibodies in two population-based studies in children and adolescents, Germany, 2003 to 2006 and 2014 to 2017", on behalf of the authors, who remain responsible for the accuracy and appropriateness of the content. The same standards for ethics, copyright, attributions and permissions as for the article apply. Supplements are not edited by *Eurosurveillance* and the journal is not responsible for the maintenance of any links or email addresses provided therein.

Variable description and categorization

The variables socio-economic status (SES), migration background and media use were categorized according to study-specific criteria and were part of the provided datasets.
The categorization of SES was based on an index, which was calculated using information regarding education and occupational qualifications, occupational status and net equivalent income of the parents. The SES score, which can be 3-21 points, is formed by summing the three subscores of education, income and occupation. The scale level is metric, the scores are rounded to one decimal place. Based on the weighted distribution of SES in the population, the index is divided into five quintiles. The lower 20% of the scores ordered by size were assigned with "low SES" (1st quintile), the middle 60% as "medium SES" (2nd to 4th quintile) and the top 20% are referred to as "high SES" (5th quintile). In KIGGS Wave 2, the creation of the SES is based on the information on the social parents (the person with whom the parent interview is conducted and his/her partner, if this person lives in the household). This is different from the written questionnaire in the KiGGS Baseline survey, where it was left up to the mother, for example, whether to provide information on the father, even if he does not live in the joint household. For more details, please see the following reference [1].
Participants were assigned having a migration status if the participant has moved to Germany and at least one parent was born abroad, if both parents have moved to Germany or if neither parent has German citizenship. Migration background was defined as study participants with migrant status or without German citizenship [2].
The variable "weekly media consumption" was created based on an index. Answers to questions on daily media consumption were converted from ordinal scale to hours using the values 0, 0.5, 1.5, 2.5, 3.5 and 5. The data on television/video/DVD, game consoles/computer games, computer/internet/smartphone were added up (DVD, computer games and smartphone were added in KiGGS2) and multiplied by 7. If data for one of the questions was missing, the number of hours was missing. This variable was created for participants from 3 years to 17 years. In KiGGS Baseline, the answer category 2–3 hours is missing in the variables assessing daily media consumption, limiting comparability between the "weekly media consumption" between KiGGS Baseline and KiGGS Wave 2. For participants 3–10 years, the parents' statements on television/video/DVD and computer/internet/smartphone were used. For participants 11–17 years, the respondents' data on television/video/DVD, computer/internet/smartphone and, in addition, game consoles/computer games were used. The three categories low, middle and high were assigned based on age-specific terciles in KiGGS Baseline and were adopted in KiGGS Wave 2.

Test systems used for serological testing

The quantitative ELISA (Enzygnost Lyme link VlsE/IgG, Siemens Healthcare Diagnostics GmbH, Eschborn, Germany) test is based on a detergent extract from cultured *B. afzelii* (strain PKo) mixed with recombinant VlsE from *Bb* s.s. (strain B31), *B. afzelii* (strain PKo), and *B. bavariensis* (strain PBi). The test was automatically processed and interpreted as recommended by the manufacturer. For each run, a cut-off was automatically calculated by the alpha method, resulting in a so-called floating cut-off that enables to determine whether a sample is negative, borderline or positive [3, 4]. This is expressed in arbitrary units (ELISA units) calculated from optical density, used synonymously to antibody titers.

The immunoblot test (Borrelia Europe plus TpN17 LINE, IgG, Virotech, Rüsselsheim, Germany) contains different antigens and tests the reactivity of sera with those. Antigens are bound to a nitrocellulose membrane. The test included the purified antigens OspC, DbpA, and p83 (all from *B. afzelii* strain PKo) and the recombinant antigens VlsE (from *Bb* s.s. strain B31 and *B. garinii* strain IP90), p58 (*B. bavariensis* strain PBi), p39 (BmpA; PKo), DbpA (from *B. garinii* strain PBr, *B. bavariensis* strain PBi, and *B. spielmanii* strain A14S). The test was performed and interpreted according to manufacturer's recommendations. Combining included DbpA antigens, the test could

detect up to six *Bb*-specific antigen reactions. As per European standards, the result of the immunoblot was considered positive if serum was reactive to at least two bands. Results are read and interpreted visually, based on a comparison with a cut-off control by two persons. Cross-reactions or reactions to non-specific antibodies play a minor role in our study, as we used a two-tier approach to test for IgG. Also, IgG are more specific as compared to IgM antibodies. Additionally, sera were tested for immune reaction to the recombinant antigen TpN17, a reaction specific to *Treponema (T.) pallidum*. Reaction to TpN17 was tested in order to exclude false-positive results through potential cross-reactivity to *T. pallidum* [5].

Test results categorization

Samples were defined seronegative when the ELISA result was negative, when a negative immunoblot followed a positive ELISA result or when a borderline or negative immunoblot result followed a borderline ELISA result. Samples were defined seropositive when a positive ELISA result was followed by a positive or borderline immunoblot result, or when a borderline ELISA result was followed by a positive immunoblot result. The final result was considered missing in case a confirmatory immunoblot test was not possible (e.g. due to the lack of serum) following a positive or borderline ELISA result (Sup Figure 1).

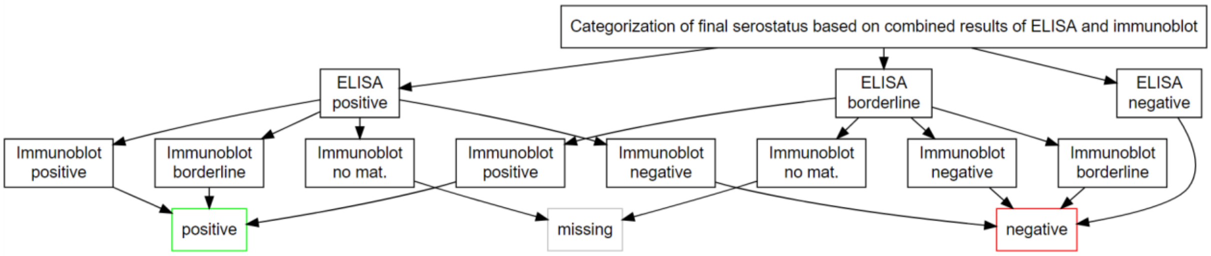

no. mat = lack of serum for testing

Figure S1: Categorization scheme to define the serostatus for *Borrelia burgdorferi* based on a two-step diagnostic approach

Causal structures and model building

Variable selection and model building was based on directed acyclic graphs (DAG) [6]. For all analyses, we used the statistical software R (version 3.6.1). Visualizations were realised using the R-package *ggplot2* [7]. To account for the study design and apply study-specific weights, we used the R-package *survey* [8]. For the DAGs, we used an R-package *dagitty* based webpage tool [9].

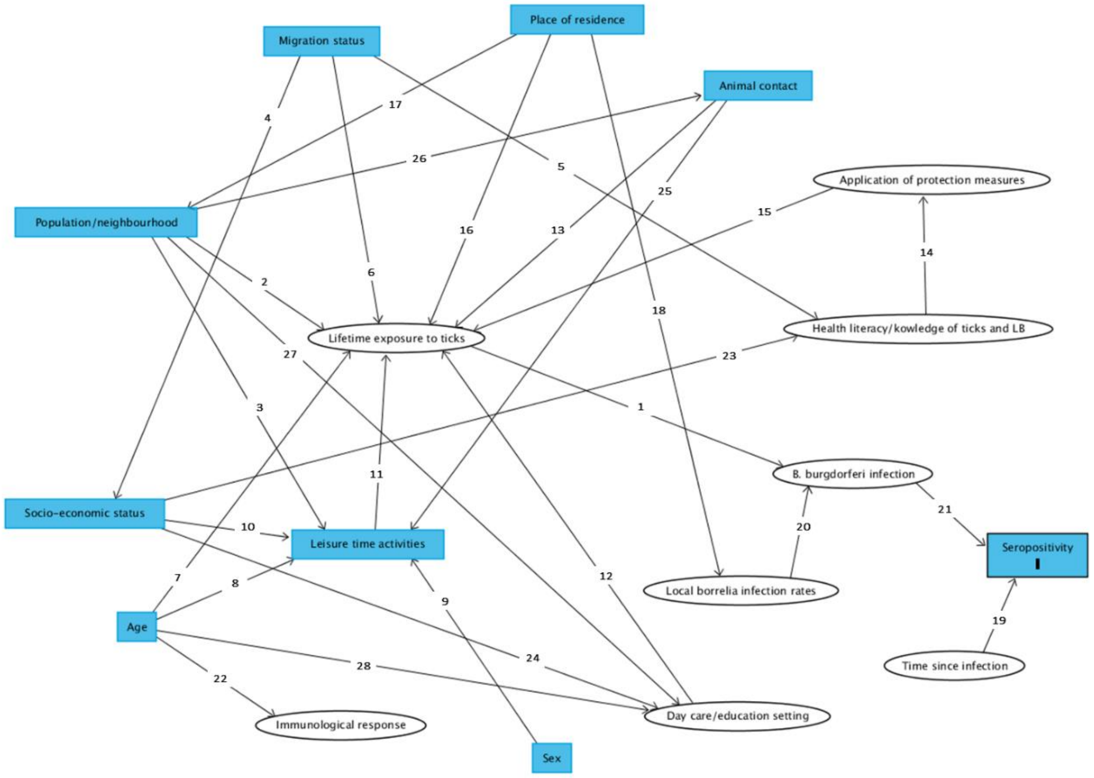

Figure S2: Directed acyclic graph (DAG) displaying hypothetical causal relationships between different variables and seropositivity for *Borrelia burgdorferi*-specific IgG antibodies (outcome of interest characterized through "I"; unmeasured variables are circled)

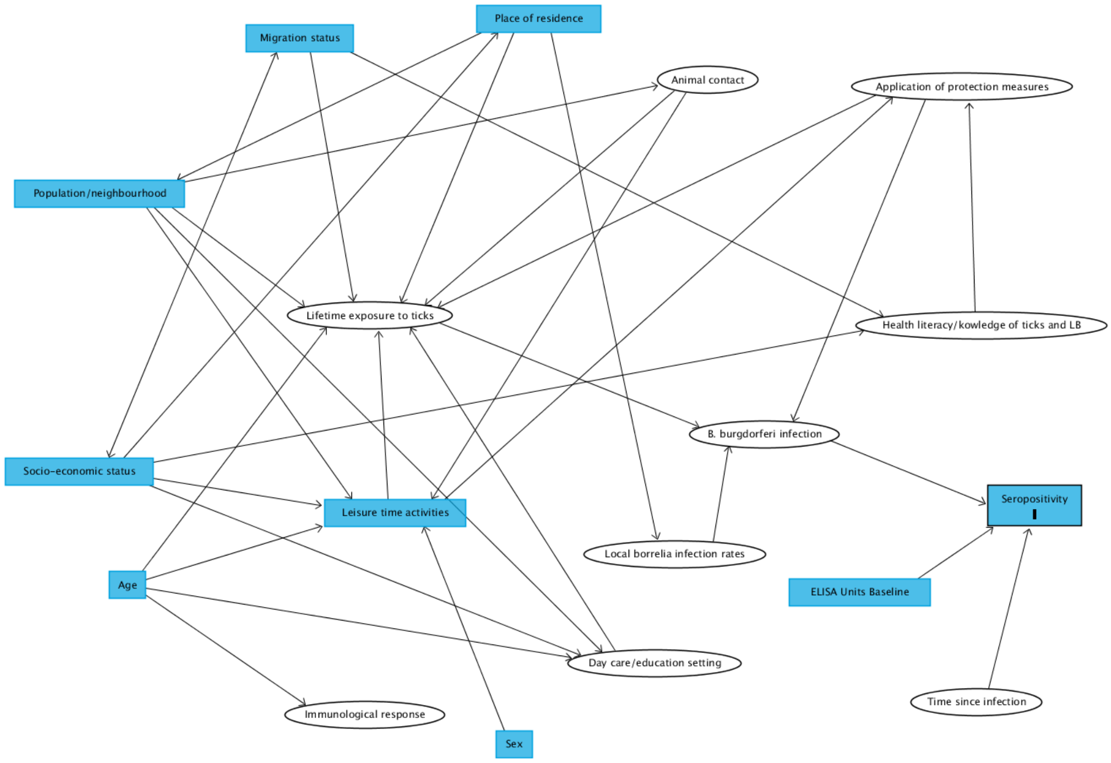

Figure S3: Directed acyclic graph (DAG) displaying hypothetical causal relationships between different variables and change of serostatus in persons seropositive for *Borrelia burgdorferi*-specific IgG antibodies (outcome of interest characterized through "I"; unmeasured variables are circled)

Table S1: Hypothesized causal relationships between different variables and seropositivity for *Borrelia burgdorferi*-specific IgG antibodies based on scientific studies and assumptions

№	Reference(s)/Assumption
**Based on scientific studies**	
1	Exposure to ticks increases the risk of tick bites and thus, risk of *Bb* infection [10, 11].
2	As living in more rural areas is often associated with increased contact to tick habitat (gardens, woodland etc.), exposure to ticks is higher [12-18].
3	Living in more rural areas is associated with certain leisure time activities, such as playing outdoors [19].
4	Migration status was found to be associated with lower SES [20].
5	Migration status was found to be associated with a lower level of health literacy [20-22], assuming that this also plays a role for specific knowledge regarding ticks and Lyme Borreliosis (LB) (potentially no contact to or knowledge of ticks/LB before migration; potential language barrier).
6	Migration status can be associated with decreased life time exposure to ticks (potentially no presence of ticks and/or LB in country of origin and therefore no contact before migration), as abundance of competent vectors is limited to certain geographic areas [23]. Migration status was found to be associated with lower seroprevalence, indicative of that [3, 14].
7	The chance of having been exposed to ticks increases with age [4, 10, 24-26].
8	Age is associated with leisure time activities; e.g. younger children are more likely to be involved in outdoor playing [27].
9	Sex is associated with leisure time activities, e.g. boys are more likely to be involved in outdoor activities than girls [19, 27].
10	SES is associated with leisure time activities, e.g. children from families with lower SES spend more time using electronic media, whereas higher SES is associated with outdoor playing [10, 19, 27-29].
11	Leisure time activities are associated with tick exposure, e.g. activities involving being outdoors increase the chance of contact to ticks [10, 14, 30, 31].
12	Frequency of playing outdoors and outdoor environments provided in care and education settings decrease towards older age (kindergarten, primary school, secondary school).<br>Certain settings with focus on outdoor activities, such as forest kindergartens, are associated with increased exposure to ticks [32].
13	Contact to animals/having a pet increases the exposure to ticks. Ticks may be attached to animals and pets, such as cats and dogs, can bring them into the home [10, 16, 33].
14	Knowledge about ticks and LB is associated with the application of prevention and protection measures [34-36].
15	The application of protection measures can help reduce the exposure to ticks, tick bites and LB [10, 12, 13, 30].
16	Abundance of ticks is heterogeneous and dependent on suitable tick habitat, increasing the risk of exposure to ticks in people living in areas within or close to suitable tick habitat [18, 37].
17	Place of residence (here we used Nielsen Areas, grouping the 16 federal states into 7 areas) is associated with community sizes and likelihood of living in more rural or urban areas, as this is heterogeneous between federal states in Germany [38].
18	*Borrelia* infection rates are heterogeneous in relation to geographical area and associated with place of residence, especially on smaller local levels [10, 18, 37, 39-42]
19	The more time has passed since infection, the more likely it is that antibodies have waned over time [43].
20	The higher the prevalence of *Borrelia* in ticks, the higher the chance of getting infected after a tick bite [44].
21	Infections, if not cleared quickly by the immune system after entering of the pathogen or due to early antibiotic treatment, lead to an immune response and result in seropositive test results, at least for some time [44].
**Based on plausible assumptions**	
22	Age is known to be associated with the immune response in individuals. Therefore, we may assume that immune reactions to an infection with *Bb* may also differ with regards to age.
23	Lower levels of education, as part of SES, may be associated with lower levels of health literacy overall [22] and less knowledge regarding ticks and LB.
24	SES may have an influence on the care or education setting, by means of choice, preferences or options.
25	Having a pet, such as a dog, or owning a horse, likely has a large impact on leisure time activities, such as going for walks and spending time outdoors.

26	Living in more rural areas is probably associated with animal contact overall, but also with owning a pet.
27	In rural areas, as compared to urban areas, day care settings are probably more likely to have a wider range of outdoor space, such as gardens/forest access, but may also have a larger focus on outdoor activities, such as forest kindergartens or Waldorf schools.
28	Age is associated with change from e.g. kindergarten to primary to secondary school; in turn, change of setting is likely associated with providing less outdoor space and secondly, children/adolescents often commute from rural areas to towns and cities, further away from the place of residence.

Table S2: Adjustment sets for seropositivity and seroconversion for *Borrelia burgdorferi*-specific IgG antibodies

Exposure	Minimal Sufficient Adjustment Set
Sex	No adjustment necessary to estimate total effect of sex on seropositivity
Age	No adjustment necessary to estimate total effect of age on seropositivity
Place of residence	No adjustment necessary to estimate total effect of place of residence on seropositivity
Population/neighbourhood	Place of residence
Socio-economic status	*Migration status*
Migration status	No adjustment necessary to estimate total effect of migration status on seropositivity
Leisure time activities	Age, *animal contact*, population/neighbourhood, socio-economic status
Animal contact	Population/neighbourhood

Variables in italics were not available in KiGGS Wave 2 and thus, were not adjusted for in the multivariable models of the KiGGS Wave 2 survey or considered for analysis regarding seroconversion

Survey and study participants

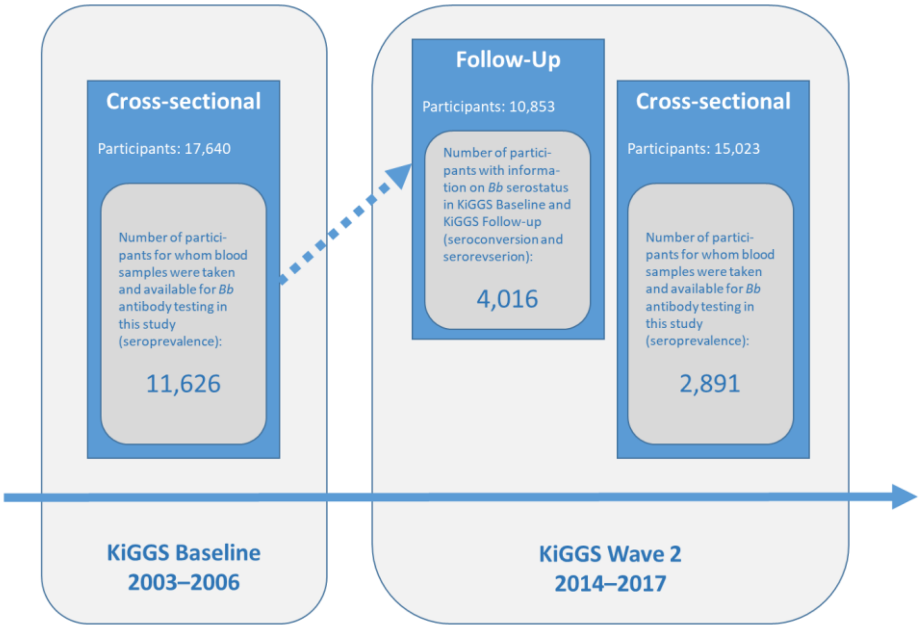

Figure S4: Overview of KiGGS Baseline and KiGGS Wave 2 surveys participants overall and number of participants included in our study

Supplementary results

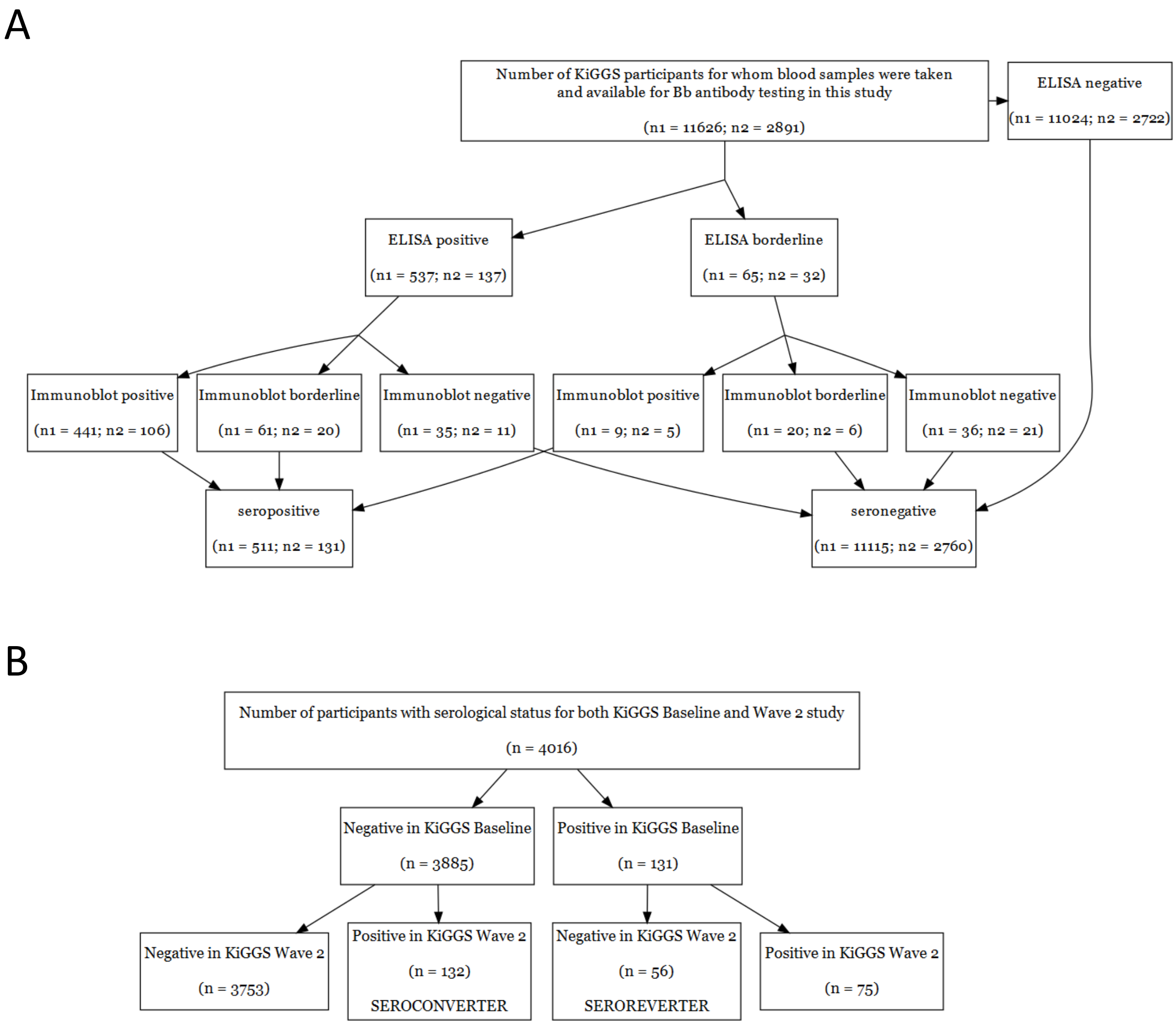

Figure S5: Categorization of Borrelia burgdorferi-specific IgG serostatus in participants of the first survey (KiGGS Baseline) (n1) and the second survey (KiGGS Wave 2) (n2) (**A**) and Borrelia burgdorferi-specific IgG serostatus in both KiGGS Baseline and KiGGS Wave 2, including seroconversions and seroreversion between the two study periods (**B**)

Table S3: Weighted IgG seroprevalence of *Borrelia burgdorferi* by age group and sex among study participants of KiGGS Baseline and KiGGS Wave 2, respectively

	Female			Male			
	KiGGS Baseline (2003–2006)						
Age group	**positive / N** (unweighted)	**IgG Seroprevalence (%)**	**95%-CI**	**positive / N** (unweighted)	**IgG Seroprevalence (%)**	**95%-CI**	**p value[a]**
3–6	34/1,102	2.54	1.50–3.58	27/1,201	2.10	1.16–3.04	0.5151
7–10	49/1,427	3.49	2.41–4.57	70/1,487	4.61	3.38–5.84	0.1778
11–13	48/1,322	2.78	1.76–3.80	71/1,368	5.07	3.64–6.50	0.0120
14–17	81/1,595	4.59	3.56–5.63	131/1,613	7.65	6.27–9.02	<0.001
	KiGGS Wave 2 (2014–2017)						
Age group							
3–6	3/367	0.40	0–0.88	8/289	2.28	0–4.57	0.0151
7–10	10/332	3.24	1.13–5.35	15/354	3.75	1.41–6.10	0.7283
11–13	15/355	3.51	1.39–5.63	25/326	5.03	2.73–7.33	0.3468
14–17	21/469	3.84	1.95–5.72	34/368	9.58	5.52–13.64	0.0054

CI=Confidence interval
[a] For comparison of seroprevalence between boys and girls, we used Pearson's chi-squared test

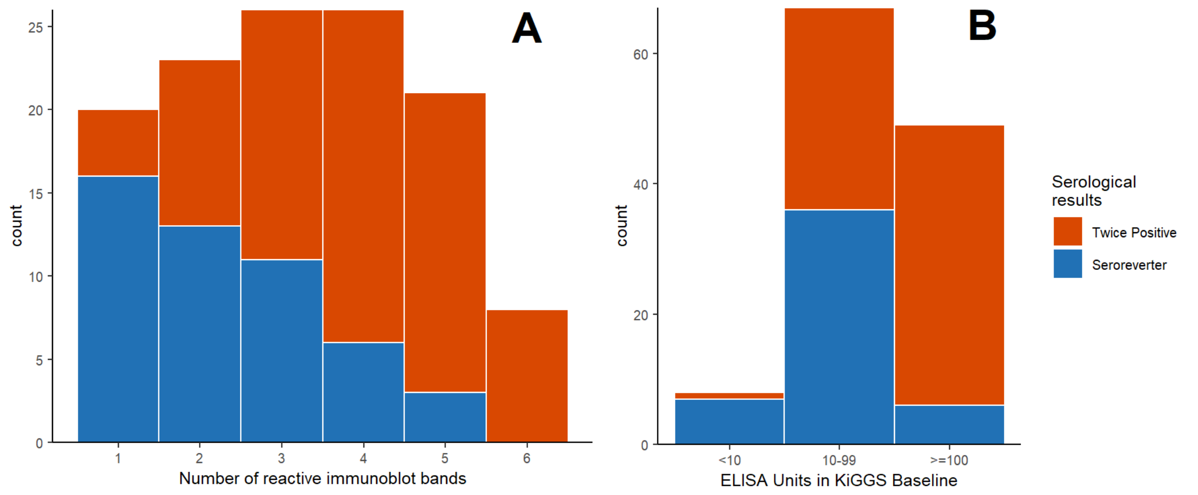

Figure S6: Borrelia burgdorferi-specific IgG serostatus of participants in KiGGS Wave 2 with initial positive serostatus in KiGGS Baseline (n=131) by the number of reactive immunoblot bands (**A**) and categorized ELISA units (**B**) in KiGGS Baseline

Supplementary references

[1] T. Lampert, J. Hoebel, B. Kuntz, S. Müters, and L. E. Kroll, "Socioeconomic status and subjective social status measurement in KiGGS Wave 2," *Journal of Health Monitoring,* vol. 3, no. 1, pp. 108-125, 2018, doi: 0.17886/RKI-GBE-2018-033.

[2] L. Schenk, U. Ellert, and H. Neuhauser, "[Children and adolescents in Germany with a migration background. Methodical aspects in the German Health Interview and Examination Survey for Children and Adolescents (KiGGS)]," *Bundesgesundheitsblatt Gesundheitsforschung Gesundheitsschutz,* vol. 50, no. 5-6, pp. 590-9, May-Jun 2007, doi: 10.1007/s00103-007-0220-z. Kinder und Jugendliche mit Migrationshintergrund in Deutschland. Methodische Aspekte im Kinder- und Jugendgesundheitssurvey (KiGGS).

[3] M. Dehnert *et al.*, "Seropositivity of Lyme borreliosis and associated risk factors: a population-based study in Children and Adolescents in Germany (KiGGS)," *PLoS One,* vol. 7, no. 8, p. e41321, 2012, doi: 10.1371/journal.pone.0041321.

[4] T. Woudenberg *et al.*, "Dynamics of Borrelia burgdorferi-Specific Antibodies: Seroconversion and Seroreversion between Two Population-Based, Cross-Sectional Surveys among Adults in Germany," *Microorganisms,* vol. 8, no. 12, Nov 25 2020, doi: 10.3390/microorganisms8121859.

[5] C. Eldin, B. Jaulhac, O. Mediannikov, J. P. Arzouni, and D. Raoult, "Values of diagnostic tests for the various species of spirochetes," *Med Mal Infect,* vol. 49, no. 2, pp. 102-111, Mar 2019, doi: 10.1016/j.medmal.2019.01.009.

[6] T. J. VanderWeele, M. A. Hernan, and J. M. Robins, "Causal directed acyclic graphs and the direction of unmeasured confounding bias," *Epidemiology,* vol. 19, no. 5, pp. 720-8, Sep 2008, doi: 10.1097/EDE.0b013e3181810e29.

[7] H. Wickham, W. Chang, and M. H. Wickham, "Package ‘ggplot2’," *Create Elegant Data Visualisations Using the Grammar of Graphics. Version,* vol. 2, no. 1, pp. 1-189, 2016.

[8] T. Lumley, "Survey: analysis of complex survey samples," 2019.

[9] J. Textor, B. van der Zander, M. S. Gilthorpe, M. Liskiewicz, and G. T. Ellison, "Robust causal inference using directed acyclic graphs: the R package 'dagitty'," *Int J Epidemiol,* vol. 45, no. 6, pp. 1887-1894, Dec 1 2016, doi: 10.1093/ije/dyw341.

[10] A. Rizzoli, H. Hauffe, G. Carpi, H. G. Vourc, M. Neteler, and R. Rosa, "Lyme borreliosis in Europe," *Euro Surveill,* vol. 16, no. 27, Jul 7 2011. [Online]. Available: https://www.ncbi.nlm.nih.gov/pubmed/21794218.

[11] K. Babu, K. R. Murthy, M. Bhagya, P. R. Murthy, V. N. Puttamallesh, and V. Ravi, "Seroprevalence of Lymes disease in the Nagarahole and Bandipur forest areas of South India," *Indian J Ophthalmol,* vol. 68, no. 1, pp. 100-105, Jan 2020, doi: 10.4103/ijo.IJO_943_19.

[12] G. Stanek, G. P. Wormser, J. Gray, and F. Strle, "Lyme borreliosis," *Lancet,* vol. 379, no. 9814, pp. 461-73, Feb 4 2012, doi: 10.1016/S0140-6736(11)60103-7.

[13] G. Smith, E. P. Wileyto, R. B. Hopkins, B. R. Cherry, and J. P. Maher, "Risk factors for lyme disease in Chester County, Pennsylvania," *Public Health Rep,* vol. 116 Suppl 1, pp. 146-56, 2001, doi: 10.1093/phr/116.S1.146.

[14] H. Wilking, V. Fingerle, C. Klier, M. Thamm, and K. Stark, "Antibodies against Borrelia burgdorferi sensu lato among Adults, Germany, 2008-2011," *Emerg Infect Dis,* vol. 21, no. 1, pp. 107-10, Jan 2015, doi: 10.3201/eid2101.140009.

[15] M. E. Killilea, A. Swei, R. S. Lane, C. J. Briggs, and R. S. Ostfeld, "Spatial dynamics of lyme disease: a review," *Ecohealth,* vol. 5, no. 2, pp. 167-95, Jun 2008, doi: 10.1007/s10393-008-0171-3.

[16] T. F. Jones, R. L. Garman, B. LaFleur, S. J. Stephan, and W. Schaffner, "Risk factors for tick exposure and suboptimal adherence to preventive recommendations," *Am J Prev Med,* vol. 23, no. 1, pp. 47-50, Jul 2002, doi: 10.1016/s0749-3797(02)00440-3.

[17] S. Mavin, P. C. Hopkins, A. MacLennan, A. W. Joss, and D. O. Ho-Yen, "Urban and rural risks of Lyme disease in the Scottish Highlands," *Scott Med J,* vol. 54, no. 2, pp. 24-6, May 2009, doi: 10.1258/rsmsmj.54.2.24.

[18] D. Hauck, A. Springer, L. Chitimia-Dobler, and C. Strube, "Two-year monitoring of tick abundance and influencing factors in an urban area (city of Hanover, Germany)," *Ticks Tick Borne Dis,* vol. 11, no. 5, p. 101464, Sep 2020, doi: 10.1016/j.ttbdis.2020.101464.
[19] M. J. Aarts, W. Wendel-Vos, H. A. van Oers, I. A. van de Goor, and A. J. Schuit, "Environmental determinants of outdoor play in children: a large-scale cross-sectional study," *Am J Prev Med,* vol. 39, no. 3, pp. 212-9, Sep 2010, doi: 10.1016/j.amepre.2010.05.008.
[20] O. Razum and J. Wenner, "Social and health epidemiology of immigrants in Germany: past, present and future," *Public Health Rev,* vol. 37, p. 4, 2016, doi: 10.1186/s40985-016-0019-2.
[21] D. Schaeffer, E. M. Berens, and D. Vogt, "Health Literacy in the German Population," *Dtsch Arztebl Int,* vol. 114, no. 4, pp. 53-60, Jan 27 2017, doi: 10.3238/arztebl.2017.0053.
[22] M. T. Svendsen *et al.*, "Associations of health literacy with socioeconomic position, health risk behavior, and health status: a large national population-based survey among Danish adults," *BMC Public Health,* vol. 20, no. 1, p. 565, Apr 28 2020, doi: 10.1186/s12889-020-08498-8.
[23] A. M. Schotthoefer and H. M. Frost, "Ecology and Epidemiology of Lyme Borreliosis," *Clin Lab Med,* vol. 35, no. 4, pp. 723-43, Dec 2015, doi: 10.1016/j.cll.2015.08.003.
[24] V. Zajac, J. Pinkas, A. Wojcik-Fatla, J. Dutkiewicz, A. Owoc, and I. Bojar, "Prevalence of serological response to Borrelia burgdorferi in farmers from eastern and central Poland," *Eur J Clin Microbiol Infect Dis,* vol. 36, no. 3, pp. 437-446, Mar 2017, doi: 10.1007/s10096-016-2813-7.
[25] M. Cora *et al.*, "Determination of Seroprevalence of Borrelia burgdorferi IgG in Adult Population Living in Trabzon," *Balkan Med J,* vol. 34, no. 1, pp. 47-52, Jan 2017, doi: 10.4274/balkanmedj.2015.0478.
[26] D. F. Vestrheim, R. A. White, I. S. Aaberge, and A. Aase, "Geographical differences in seroprevalence of Borrelia burgdorferi antibodies in Norway, 2011-2013," *Ticks Tick Borne Dis,* vol. 7, no. 5, pp. 698-702, Jul 2016, doi: 10.1016/j.ttbdis.2016.02.020.
[27] L. Auhuber, M. Vogel, N. Grafe, W. Kiess, and T. Poulain, "Leisure Activities of Healthy Children and Adolescents," *Int J Environ Res Public Health,* vol. 16, no. 12, Jun 12 2019, doi: 10.3390/ijerph16122078.
[28] J. D. Finger, G. B. Mensink, W. Banzer, T. Lampert, and T. Tylleskar, "Physical activity, aerobic fitness and parental socio-economic position among adolescents: the German Health Interview and Examination Survey for Children and Adolescents 2003-2006 (KiGGS)," *Int J Behav Nutr Phys Act,* vol. 11, no. 1, p. 43, Mar 22 2014, doi: 10.1186/1479-5868-11-43.
[29] A. I. Wijtzes *et al.*, "Increased risk of exceeding entertainment-media guidelines in preschool children from low socioeconomic background: the Generation R Study," *Prev Med,* vol. 55, no. 4, pp. 325-329, Oct 2012, doi: 10.1016/j.ypmed.2012.07.023.
[30] C. Finch *et al.*, "Integrated assessment of behavioral and environmental risk factors for Lyme disease infection on Block Island, Rhode Island," *PLoS One,* vol. 9, no. 1, p. e84758, 2014, doi: 10.1371/journal.pone.0084758.
[31] L. Stjernberg and J. Berglund, "Risk of acquiring tick bites in south-eastern Sweden," *Scand J Infect Dis,* vol. 34, no. 11, pp. 840-4, 2002, doi: 10.1080/0036554021000026955.
[32] E. Weisshaar, A. Schaefer, R. R. Scheidt, T. Bruckner, C. J. Apfelbacher, and T. L. Diepgen, "Epidemiology of tick bites and borreliosis in children attending kindergarten or so-called "forest kindergarten" in southwest Germany," *J Invest Dermatol,* vol. 126, no. 3, pp. 584-90, Mar 2006, doi: 10.1038/sj.jid.5700160.
[33] E. H. Jones *et al.*, "Pet ownership increases human risk of encountering ticks," *Zoonoses Public Health,* vol. 65, no. 1, pp. 74-79, Feb 2018, doi: 10.1111/zph.12369.
[34] C. Aenishaenslin, C. Bouchard, J. K. Koffi, and N. H. Ogden, "Exposure and preventive behaviours toward ticks and Lyme disease in Canada: Results from a first national survey," *Ticks Tick Borne Dis,* vol. 8, no. 1, pp. 112-118, Jan 2017, doi: 10.1016/j.ttbdis.2016.10.006.
[35] D. J. Beaujean, M. Bults, J. E. van Steenbergen, and H. A. Voeten, "Study on public perceptions and protective behaviors regarding Lyme disease among the general public in the Netherlands: implications for prevention programs," *BMC Public Health,* vol. 13, p. 225, Mar 15 2013, doi: 10.1186/1471-2458-13-225.

[36] F. Mowbray, R. Amlot, and G. J. Rubin, "Predictors of protective behaviour against ticks in the UK: a mixed methods study," *Ticks Tick Borne Dis,* vol. 5, no. 4, pp. 392-400, Jun 2014, doi: 10.1016/j.ttbdis.2014.01.006.

[37] A. Schwarz, W. A. Maier, T. Kistemann, and H. Kampen, "Analysis of the distribution of the tick Ixodes ricinus L. (Acari: Ixodidae) in a nature reserve of western Germany using Geographic Information Systems," *Int J Hyg Environ Health,* vol. 212, no. 1, pp. 87-96, Jan 2009, doi: 10.1016/j.ijheh.2007.12.001.

[38] P. Küpper, "[Delimitation and typification of rural areas]," in "Thünen Working Paper 68," Thünen-Institut für Ländliche Räume, Braunschweig, Germany, 2016, vol. Thünen Working Paper 68.

[39] M. B. Vögerl, "[Ticks and tick-borne infectious diseases in Lower Bavaria and the Upper Palatinate: Prevalence of B. burgdorferi s.l. in I. ricinus in Lower Bavaria and the Upper Palatinate]," Tierärztlichen Fakultät der Ludwig-Maximilians-Universität München, Ludwig-Maximilians-Universität München, 2013.

[40] D. Galfsky, A. Obiegala, N. Król, and M. Pfeffer, "[Tick-borne pathogens in small mammals and ticks from Saxony]," presented at the Süddeutscher Zeckenkongress, 2018.

[41] D. Zubrikova *et al.*, "Prevalence of tick-borne encephalitis virus and Borrelia burgdorferi sensu lato in Ixodes ricinus ticks in Lower Bavaria and Upper Palatinate, Germany," *Ticks Tick Borne Dis,* vol. 11, no. 3, p. 101375, May 2020, doi: 10.1016/j.ttbdis.2020.101375.

[42] M. Okeyo *et al.*, "Longitudinal study of prevalence and spatio-temporal distribution of Borrelia burgdorferi sensu lato in ticks from three defined habitats in Latvia, 1999-2010," *Environ Microbiol,* vol. 22, no. 12, pp. 5033-5047, Dec 2020, doi: 10.1111/1462-2920.15100.

[43] R. A. Kalish, G. McHugh, J. Granquist, B. Shea, R. Ruthazer, and A. C. Steere, "Persistence of immunoglobulin M or immunoglobulin G antibody responses to Borrelia burgdorferi 10-20 years after active Lyme disease," *Clin Infect Dis,* vol. 33, no. 6, pp. 780-5, Sep 15 2001, doi: 10.1086/322669.

[44] J. D. Radolf, K. Strle, J. E. Lemieux, and F. Strle, "Lyme Disease in Humans," *Curr Issues Mol Biol,* vol. 42, pp. 333-384, 2021, doi: 10.21775/cimb.042.333.
